# Alendronate-induced Perturbation of the Bone Proteome and Microenvironmental Pathophysiology

**DOI:** 10.7150/ijms.61552

**Published:** 2021-07-23

**Authors:** Jayoung Kim, Austin Yeon, Sarah J. Parker, Muhammad Shahid, Aissatou Thiombane, Eunho Cho, Sungyong You, Hany Emam, Do-Gyoon Kim, Minjung Kim

**Affiliations:** 1 Departments of Surgery and Biomedical Sciences, Cedars-Sinai Medical Center, Los Angeles, CA, USA; 2 Department of Medicine, University of California Los Angeles, CA, USA; 3 Smidt Heart Institute, Department of Cardiology, Cedars-Sinai Medical Center, Los Angeles, CA, USA; 4 Division of Orthodontics, College of Dentistry, The Ohio State University, Columbus, OH, USA; 5 Division of Oral Surgery, College of Dentistry, The Ohio State University, Columbus, OH, USA; 6 Department of Cell Biology, Microbiology, and Molecular Biology, University of South Florida, Tampa, FL, USA

**Keywords:** Osteonecrosis of the jaw, bisphosphonate, GSK signaling, clinical cone beam computed tomography, bone mineral density, proteomics, biomarker

## Abstract

**Objectives:** Bisphosphonates (BPs) are powerful inhibitors of osteoclastogenesis and are used to prevent osteoporotic bone loss and reduce the risk of osteoporotic fracture in patients suffering from postmenopausal osteoporosis. Patients with breast cancer or gynecological malignancies being treated with BPs or those receiving bone-targeted therapy for metastatic prostate cancer are at increased risk of bisphosphonate-related osteonecrosis of the jaw (BRONJ). Although BPs markedly ameliorate osteoporosis, their adverse effects largely limit the clinical application of these drugs. This study focused on providing a deeper understanding of one of the most popular BPs, the alendronate (ALN)-induced perturbation of the bone proteome and microenvironmental pathophysiology.

**Methods:** To understand the molecular mechanisms underlying ALN-induced side-effects, an unbiased and global proteomics approach combined with big data bioinformatics was applied. This was followed by biochemical and functional analyses to determine the clinicopathological mechanisms affected by ALN.

**Results:** The findings from this proteomics study suggest that the RIPK3/Wnt/GSK3/β-catenin signaling pathway is significantly perturbed upon ALN treatment, resulting in abnormal angiogenesis, inflammation, anabolism, remodeling, and mineralization in bone cells in an *in vitro* cell culture system.

**Conclusion:** Our investigation into potential key signaling mechanisms in response to ALN provides a rational basis for suppressing BP-induced adverse effect and presents various therapeutic strategies.

## Introduction

Bone tissue undergoes continuous cycles of bone resorption by osteoclasts and bone formation by osteoblasts, which were orchestrated by osteocytes^1^. Bone tissue is also highly vascularized providing O_2_, nutrients, and precursor cells for bone remodeling and serving as routes for blood and immune cells into bone tissue. Regulatory interactions between cells of these hematopoietic, immune, and skeletal (bone) systems closely regulate bone remodeling and repair processes via secreted factors such as VEGF, M-CSF, RANKL, Wnt3a, and Osteoprotegerin, etc. and their cell surface receptors.

Several key signal pathway has been shown to play pivotal roles in bone remodeling/repair processes, enhancing osteoblast differentiation and angiogenesis and modulating immune cell functions^2^. Specifically, Wnt pathway activation via GSK3 inactivation leads to osteoblast differentiation and stimulates bone anabolism while GSK3 gain-of-function promotes osteogenesis of adipose-derived stromal cells, making GSK3 as a possible therapeutic target for bone diseases 3-5. Mice expressing constitutively active GSK3β (GSK3β S9A) mutant, exhibited a marked increase in osteogenesis, whereas ones with catalytically inactive GSK3β (GSK3β K85A) showed decreased osteogenic differentiation by regulating β-catenin^5^. Wnt/GSK3/β-catenin pathway also plays important roles in angiogenesis and vasculogenesis, supporting wound healing and regeneration of oral mucosa and jaw tissue 6. Wnt signaling activation by Wnt1, VEGF, or CHIR99021 (GSK3β inhibitor) enhanced, while its inactivation by JW67 (targeting APC/GSK3/β-catenin complex) or β-catenin kinase dead form suppressed, vascular differentiation of mesenchymal stem cells (MSCs) derived from dental pulp 7. GSK3β regulates β-catenin level in endothelial cells. Expression of β-catenin in HUVEC cells increases VEGF-A and -C level and induces capillary formation 8.

Bisphosphonates (BPs) have been suggested to modulate the proliferation and differentiation rates of osteoblasts and trigger survival signaling leading to bone homeostasis and antiresorptive effect 9-11. First approved by the FDA in 1995, alendronate (ALN) is currently one of the most used BPs in the medical field^12^. ALN has been used successfully for the treatment of osteoporosis 13. Several pieces of evidence indicate that there is a strong association between ALN and lower risk of bone metastases in postmenopausal women with early breast cancer 14, 15. Cancer patients undergoing BPs-based treatments are at a 10-fold greater risk of developing bisphosphonate-related osteonecrosis of the jaw (BRONJ) 16, which is suggested to be a result of osteoclast inhibition and apoptosis^17^. Due to the prevalent usage of BPs in many bone-related diseases, more understanding on underlying mechanisms of adverse effect caused by BPs is crucial in providing better care and improving patient quality of life 18. In oncology patients, incidence of BRONJ has been estimated to be as high as 18.6%^19^, and risk of developing BRONJ increases with longer duration or higher dosages of BPs-based therapy^20^.

This study sought to understand the pathogenesis of BP-associated adverse effects by looking into proteome perturbation and potential molecular biomarkers and mechanisms using an *in vitro* cell culture system.

## Materials and Methods

### Reagents and cell culture

Several cell lines, including MG-63, SCC-9, SCC-15, and HUVEC cells, were obtained from the American Type Culture Collection (ATCC) (Manassas, VA). Culture condition, antibodies and reagents used for this study are available in Supplementary Materials.

### Quantitative proteomics

Sample preparation methods for this study are available in Supplementary Materials. For protein quantification and statistical analysis, mapDIA was used. Data was analyzed based on the established workflows previously described 21, 22. Briefly, peptides were identified using the openSWATH workflow 23, searched against the pan human library 24 with decoy sequences appended for false discovery rate calculation using the pyprophet algorithm 25. Peptides with no greater than 5% identified false discovery rate (FDR) across all samples were compiled into the final experimental results using the TRIC alignment algorithm 26. Following removal of non-proteotypic peptides (e.g., sequences matching more than one gene product from the Pan Human Library), the final aligned results were analyzed using mapDIA to select only high-quality performing fragments for quantification and to compile fragment level data into peptide and protein level abundance estimates 27. The mapDIA software was also used to perform pairwise comparisons between ALN and control groups, including adjustment for multiple testing effects to produce a comparison FDR, which filtered proteins with significant or non-significant differential abundance in response to ALN treatment. The MS proteomics data has been deposited to the PRIDE repository with the dataset identifier, PXD024585.

### Identification of differentially expressed proteins (DEPs)

Proteins with more than 3 nonredundant peptides in each sample were selected. Further selection of proteins detected in at least 2 samples in the same group was performed for statistical testing. A median difference test and Welch's t-test were performed separately, and the resulting two p-values were combined to compute adjusted p-values using Stouffer's method. The DEPs were identified based on an adjusted p-values<0.05 and absolute log_2_ fold-change (FC) ≧0.58.

### VEGF ELISA assay

To determine vascular endothelial growth factor (VEGF-A) levels of conditioned medium from MG-63 cells incubated with ALN, supernatants from cell cultures were analyzed using the Human VEGF Quantikine ELISA Kit (R&D Systems, Minneapolis, Mass).

### Cytokine array

Cell lysates and conditioned media from RAW 264.7 macrophages were collected and analyzed using a cytokine array, per standard provided protocols (R&D Systems, Minneapolis, MN, USA). ImageJ was used to measure the signal intensities.

### Mineralization assay using Alizarin Red-S staining

The formation of calcium phosphate was quantified in MG-63 bone cells via Alizarin Red-S mineralization assay. Optical density was detected at an absorbance of 562 nm.

### Statistical analysis

Most of the experiments were repeated at least six (6) times with independent treatments, while all the cases were repeated at least three times. Each of the experiments did not show significantly different results across replications. Statistical analyses were conducted using GraphPad Prism, version 7.03 (GraphPad Software Inc., La Jolla, CA). Mean values from technical replicates were used for statistical analyses, and all data were presented as the mean ± standard deviation (SD). A one-way analysis of variance (ANOVA) or Student's t-test was conducted to compare the groups of data. Differences were considered statistically significant when P < 0.05.

## Results

### Comprehensive analysis with large unbiased global proteomic assays suggested perturbed proteins in response to BP in bone cells

Mass spectrometry (MS) has several important attributes that make it amenable to providing reproducible and accurate assays for proteins and metabolites. It provides a scalable number of analytes quantified in a single assay and absolute quantification, which leads to a standardized path from assay development to validation of new candidate biomarkers applicable in any clinical chemistry laboratory. To understand the molecular mechanisms underlying specific diseases, an unbiased and global omics approach combined with big data analysis using bioinformatics is critical.

**Figure 1:**
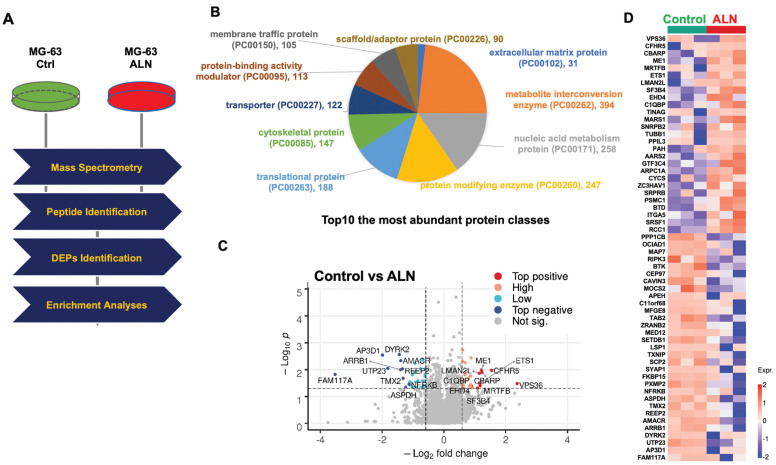
** Proteomics profiling revealing alendronate (ALN)-induced protein alteration in the global proteome of MG-63 bone cells. (A)** Experimental mass spectrometry (MS) workflow for this study. **(B)** Top 10 most abundant protein classes. **(C)** Volcano plot shows DEPs. **(D)** Heatmap depicts the differential expression patterns of proteins in response to ALN. Red and blue dots represent upregulated and downregulated proteins, respectively. Per row z-score of protein intensity is calculated. Each dot represents one protein. Proteins used are identical with those in the volcano plot. Experiments were done in triplicate.

As described in the Materials and Methods, a proteomics approach was implemented (**Fig 1A**). The top 10 most abundant protein classes are shown in **Fig 1B**. Global proteomics analysis identified a highly confident and comprehensive list of perturbed proteins in MG-63 bone cells treated with ALN. Protein quantification and statistical analysis using mapDIA identified perturbed proteins in MG-63 cells treated with 10 μM ALN. A total of 2,865 proteins with UniProtKB IDs were identified. Further analysis with the PANTHER Protein Classification Tool revealed that the most abundant top 10 proteins classes included extracellular matrix, metabolite interconversion, nucleic acid metabolism, protein modification, translational regulation, cytoskeletal, transporter, protein-binding activity modulator, membrane traffic, and scaffold/adaptor^28^. To identify DEPs, the integrated hypothesis testing method was applied. Briefly, the median difference test and Welch's t-test was performed on high confidence proteins, which in the case of this experiment, were proteins detected with more than 3 non-redundant peptides encompassing at least 2 samples in the same group. The median test p-value and Welch's t-test p-value were then combined to adjust for multiple testing errors. Finally, 27 up- and 31 downregulated DEPs were selected for based on adjusted p-values < 0.05 and log2 FC ≧0.58. Significant expression was assessed using a volcano plot (**Fig 1C and Fig 1D**) and heatmap (**Fig 1E**). The DEPs are listed in **Table 1**.

### Angiogenesis alteration in response to ALN treatment

**Figure 2:**
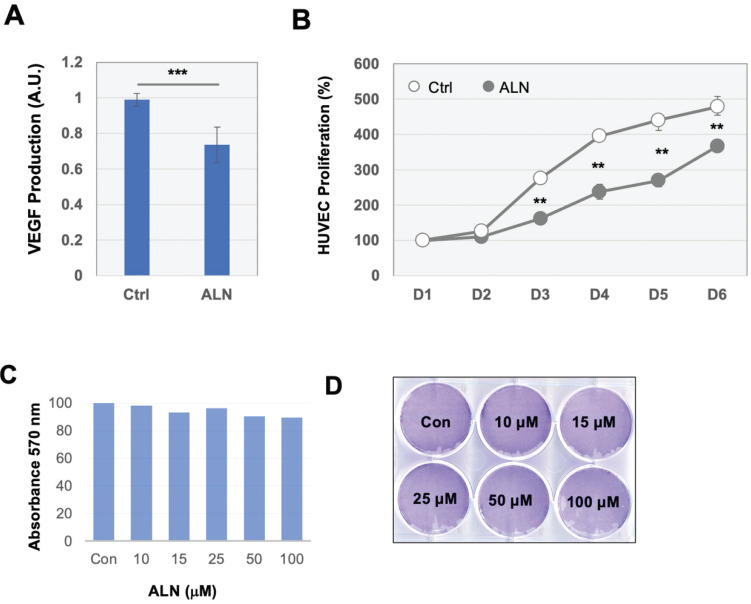
** Angiogenic pathways may be upregulated by ALN treatment. (A)** Secretion of VEGF in MG-63 bone cells treated with ALN. Effect of ALN treatment on the secreted VEGF levels into conditioned medium by MG-63 cells. Values (mean and standard deviation (SD)) are expressed as fold-changes compared to untreated cells (Ctrl, control). **(B)** Proliferation of HUVEC in the collected media of MG-63 cells. **p < 0.001, compared to control (Student's t-test).** (C-D)** No apoptosis was observed within the treatment period of 6 h. **(C).** Cell viability of MG-63 cells. MTT assay revealed no viability changes by ALN treatment.** (D)** Crystal violet staining assay showed no cell mass changes in response to varying concentration of ALN for 6 days. Experiments were done 6 times. Representative images were shown.

When verifying proteins associated with angiogenesis-related Gene Ontology Biological Processes (GOBPs), several proteins were identified, including ETS proto-oncogene 1 (ETS1) (log_2_ FC, 1.1566), integrin subunit alpha 5 (ITGA5) (log_2_ FC, 0.6102), and milk fat globule-EGF factor 8 (MFGE8) (log_2_ FC, -0.7468) (**Table 1**). To further investigate these findings, the effects of ALN on several well-known angiogenic factors were investigated. Secretion of VEGF-A, a potent angiogenic factor, was examined in bone cells after stimulation with ALN. Consistent with similarly designed work from previous trials 29, treatment of MG-63 cells with ALN led to a statistically significant but modest decrease (approximately 30%) of VEGF secretion into the conditioned medium compared to control (**Fig 2A**). Furthermore, HUVEC stimulation in the collected culture medium also exhibited modest but meaningful suppression of proliferation (**Fig 2B**). Collectively, the reduction of VEGF secretion and HUVEC proliferation by ALN strongly implies angiogenic signals to vessel cells from bone cells. This finding suggests the potential microenvironment-level regulation of bone remodeling in ONJ. For proteomics profiling, necrotic and apoptotic conditions were avoided to fully investigate the effects of ALN on bone cells. Additional analysis confirmed that there was no induced cell death with ALN treatment in MG-63 cells. Cell viability and proliferation rates, which were determined using MTT (**Fig 2C**) and crystal violet staining assays (**Fig 2D**), showed no cytotoxicity.

### Receptor-interacting protein kinase 3 (RIPK3), a necroptosis factor, is altered in the ALN-treated proteome

**Figure 3:**
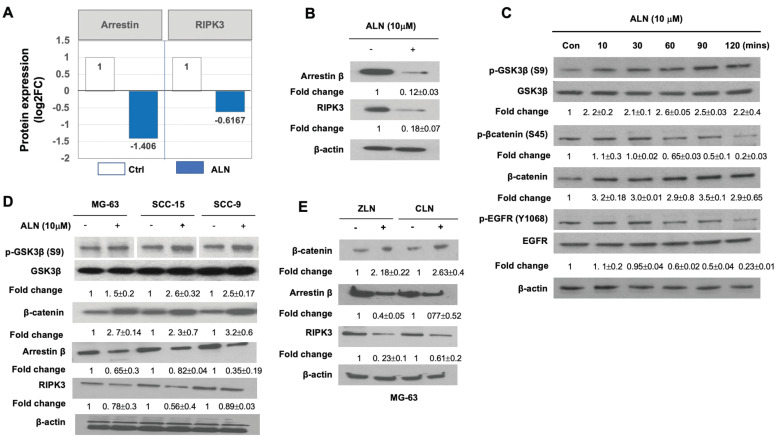
** The RIPK3/arrestin/GSK3β/ β-catenin/VEGF pathway is altered by ALN treatment. (A-B).** Quantification results showed that arrestin β and RIPK3 are significantly suppressed with ALN treatment. **(A)** Data from proteomics profiling. DEP levels obtained from proteomics analysis are shown in Table 1. **(B)** Western blot analysis to measure the expression levels of arrestin β and RIPK3 proteins in the presence or absence of ALN. β-actin was used as the loading control. **(C)** ALN-induced phosphorylation of GSK3β (S9) and β-catenin (S45) led to stabilization of β-catenin in MG-63 cells. **(D)** Comparison of phosphorylation of GSK3β and expression of β-catenin, arrestin β, and RIPK3 in MG-63, SCC-15, and SCC-9 cells after treatment with ALN. **(E)** Effects of several BPs (ZLN and CLN) on β-catenin, arrestin β, and RIPK3 in MG-63 cells. After stimulation with 10 μM of ALN, ZLN, or CLN at various times, cells were harvested for protein extraction and western blot analysis. Representative western blot images were selected after experiments were repeated 6 times.

Among the DEPs regulated by ANL treatment, proteins involved in angiogenesis, inflammation, and necrosis were of particular interest due to their relevance in ONJ. Proteomics profiling revealed downregulation of RIPK3 in MG63 cells treated with ANL (**Fig 3A**). RIPK3 has recently been reported as a mediator of necroptosis, programmed non-apoptotic cell death, and necroinflammation in response to immune signaling and cytokines, such as TNF-α 30. The inhibition of RIPK3 activity suppressed *Enterococcus faecalis* infection-induced cell death in MG-63 cells^31^. RIPK3 expression is inhibited by hypoxia, which contributes to angiogenesis 32. Loss of RIPK3 leads to the activation of the Wnt/β-catenin signaling pathway in the *ripk3*^-/-^ colon cancer mouse model, and enhances inflammation, immune cell infiltration, and angiogenesis 33.

Western blot analysis was able to validate that the protein expression levels of arrestin β1 (ARRB1) was significantly diminished by ALN treatment (**Fig 3B**), which was consistent with proteomics analysis. Given that ARRB1 is reported as a necessary component for Wnt/β-catenin signaling and as a regulator of GSK-3β activation/inactivation 34, the effects of ALN and ARRB1 on the Wnt/GSK3/β-catenin signaling cascades were another point of interest. Proteomics profiling and biochemical analysis revealed the downregulation of RIPK3 and ARRB1 by ALN treatment, which suggests that the effects of ALN on MG-63 cells are likely to be mediated by the Wnt/GSK3/β-catenin signaling pathway.

### The glycogen synthase kinase 3 (GSK3) network is an ALN regulatory signaling pathway

To understand the activation of signaling cascades in response to BP treatment in bone cells, the phosphorylation of important signaling proteins in MG-63 cells treated with ALN was assessed. The involvement of Wnt/GSK3/β-catenin signaling aberration was first determined, and the downstream secreted effectors of the Wnt pathway were evaluated as a part of the ALN signaling pathway.

Based on previous findings in literature, the Wnt/GSK3/β-catenin pathway has been shown to play a pivotal role in bone remodeling/repair processes, enhancement of osteoblast differentiation, angiogenesis, and modulation of immune cell functions^2^. This evaluation further suggests that the Wnt/GSK3/β-catenin pathway may play a key role in the biological effects of response to ALN treatment in MG-63 cells.

After treatment with ALN at varying incubation times (0, 20, 30, 60, 90, and 120 min), the phospohorylation status of a series of crucial signaling molecules was evaluated using western blot analysis. The phospohorylation of GSK-3β (S9) increased with ALN treatment (**Fig 3C**). GSK-3, a serine/threonine protein kinase that phosphorylates and inactivates glycogen synthase, is a key downstream regulator of the PI3K/Akt pathway. GSK-3 signaling is inactivated by phosphorylation of Ser9 in GSK-3β. Since the phospohorylation of GSK-3β (S9) increased, this suggests that ALN treatment inactivates GSK-3 signaling in MG-63 cells.

As an important downstream effector of the Wnt signaling pathway, β-catenin is phosphorylated at S45 by a complex of axin and casein kinase I (CKI), which initiates the β-catenin phosphorylation-degradation cascade 35. While the phospohorylation of GSK-3β (S9) increased with ALN treatment, phosphorylation of β-catenin (S45) and EGFR (Y1068) decreased (**Fig 3C**). The decreased phosphorylation of β-catenin may increase protein stability and protein expression (**Fig 3B**). Increased phosphorylation of GSK-3β (S9) was consistently observed in other cells, including SCC-9 and SCC-15, with ALN, zoledronic acid (ZLN), or clodronate (CLN) treatment (**Fig 3D and Fig 3E**). These results suggest that ANL suppresses ARRB1, inactivates GSK-3β, and stabilizes β-catenin. The RIPK3/arrestin/Wnt/GSK/β-catenin network may be a potential molecular regulatory network whose activation is altered upon ALN therapy.

### Cytokine production and secretion in RAW 264.7 macrophages may be enhanced by ALN treatment

**Figure 4:**
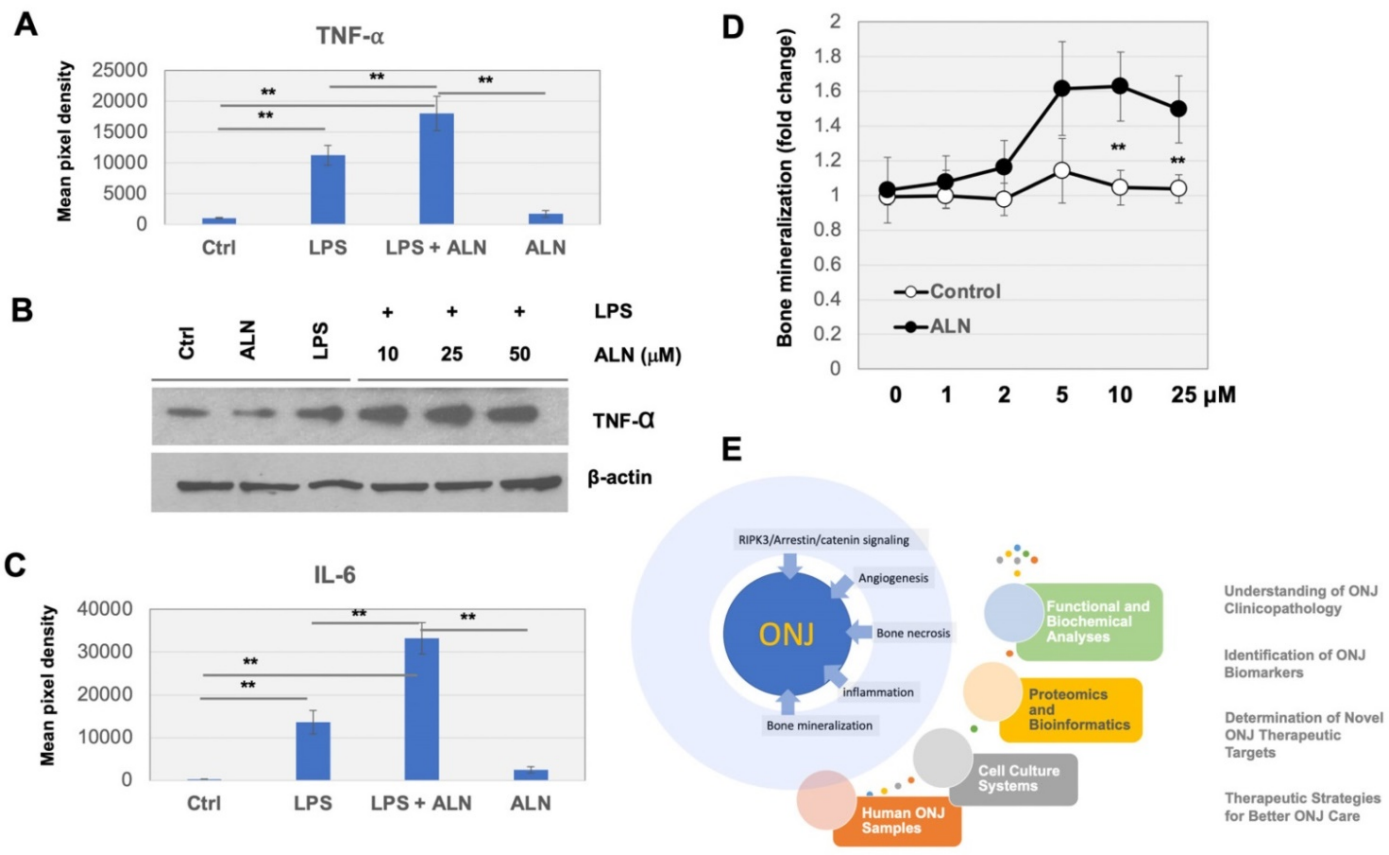
** Pro-inflammatory cytokines are produced and secreted in response to ALN treatment in RAW 264.7 macrophage cells. (A-B)** Cytokine array was conducted as described in Materials and Methods. Production of TNF-α **(A)** and secretion of IL-6 **(B)** increased with ALN treatment. **(C)** Western blot analysis for further validation. ***p < 0.001 and **p < 0.001, compared to control (Student's t-test). Representative images are shown. **(D)** ALN treatment impaired homeostasis in bone mineralization. Quantification of mineral deposition by Alizarin Red-S staining shown as a graph. Data represent average±SD (n= 6). Statistical analysis was compared between ALN and vehicle only (ctrl) (p-value<0.05).

To test the effects of ALN on the immune system, a commercially available cytokine array was used to screen for potentially stimulated cytokines. RAW 264.7 macrophages were incubated with ALN both with and without the presence of lipopolysaccharides (LPS) (100 ng/ml) for 24 h. As shown in **figure 4A**, the production of tumor necrosis factor alpha (TNF-α) was stimulated by LPS and the levels of TNF-α were significantly increased with ALN. Western blot analysis also supported these findings (**Fig 4B**). The secretion of IL-6 also greatly increased with ALN (**Fig 4C**). However, there were no dramatic additional effects across other cytokines.

### Abnormalities in calcium phosphate formation in bone cells and bone mineral density (BMD) distribution in ONJ-associated osteonecrosis

ALN is regularly used to help osteoporosis patients with bone mineralization loss. To test the effects of ANL on the quantification of mineral deposition, Alizarin Red-S staining assays were used to further assess mineralization levels after treatment. MG-63 cells were incubated with ALN or vehicle control (0, 1, 5, 10, and 25 μM) for 2 days. Incubation of cells with ALN led to a marked increase in mineralization (to ~1.6 fold) compared to controls (**Fig 4D**).

## Discussion

Our proteomics profiling revealed the downregulation of RIPK3 in response to ALN treatment in MG-63 bone cells. RIPK3 has been reported to play a fundamental role in inhibiting inflammation and mediating necroptosis and necroinflammation through the RIPK3-MLKL (mixed lineage kinase domain-like protein) pathway 30. Inhibitors of RIPK3 and MLKL suppressed cell death from *Enterococcus faecalis* infection in MG-63 cells^31^. Although not encompassed in the current study, the role, and mechanisms of RIPK3 and its downstream signaling cascades in ALN-induced bone biology are under further investigation by our group. In addition, this study showed that the presence of ALN enhanced production or secretion of inflammatory cytokines in LPS-activated macrophage cells. A previous study found that ZLN, a potent BP, stimulated and increased inflammatory osteoclastic mediators 36. Furthermore, ZLN was found to suppress proliferation and migration of vascular endothelial cells 37. Expression of VEGF receptor 2 in vascular endothelial cells was also reported in response to treatment with ZLN^38^. In our experimental system, we observed modest decreases in VEGF secretion in response to ALN treatment.

The experimental data further suggested the potential role of the Wnt/GSK3/β-catenin signaling pathway in the BP-perturbated proteome and its effects on bone homeostasis. This study demonstrated that the Wnt/GSK3/β-catenin signaling pathways may play a fundamental role in bone metabolism, homeostasis, and remodeling. Multifaceted roles of GSK3 under each cellular context have been reported. In cytotoxic T lymphocytes (CTL), GSK3 inhibition blocks programmed cell death protein-1 (PD-1) transcription; thereby, enhancing CTL functioning 39. GSK3 is a serine/threonine kinase that regulates Wnt/β-catenin, PI3K/PTEN/AKT, RAS/RAF/MAPK, hedgehog, Notch, and other signaling pathways and has been implicated in multiple diseases 40, 41. Phosphorylation of GSK-3α/β at multiple serine and threonine sites inactivates the kinase, while Tyr279/216 phosphorylation (pY) activates the kinase. GSK3 is reported to have both tumor promoting (glioblastoma, pancreatic, ovarian, and blood cancers) and tumor suppressive (breast and skin cancers) roles^42^. GSK3 stabilizes anti-apoptotic Bcl2, Bcl2L12A, c-Myb, Mcl-1, and VEGF, promoting tumors. On the other hand, GSK3 phosphorylates and destabilizes β-catenin leading to the downregulation of c-Myc and cyclin D1. GSK3 also phosphorylates T286 on cyclin D1, leading to its nuclear export and degradation^43^. Consistent with this study, previous findings have suggested an important role for the Wnt/GSK-3 signaling pathway in osteogenesis; inhibition of Wnt/GSK-3 activity induced osteoblast differentiation and significantly increased BMD in an ovariectomized rat model 44.

Experimental observation from this study suggests that a systematic overview of changes in the microenvironmental landscape is important for understanding ALN-induced pathophysiology in bone cells (**Fig 4E**). Treatment with ALN also leads to alterations in bone mineralization, which may further impair bone biology. In ONJ patients, our previous studies quantifying bone density and mineralization found that cone-beam computed tomography (CBCT) and micro-computed tomography image-based histomorphometric evaluation may be an efficient method to check bone health^45^. Abnormal BMD distribution in ONJ-associated osteonecrosis was observed by clinical CBCT imaging^46^. It would be worthwhile to determine if the patterns and severity of abnormal mineralization densities within jaw-bone biopsy samples can be implemented in ONJ patient care.

Collectively, the main innovative deliverables from this study are expected to lead to a better understanding of the mechanisms underlying ALN-induced pathological effects on bone and immune cells. The findings in this paper are promising but have several limitations; (1) the effects of BPs on osteoblast function are throughout the skeleton, and (2) ALN targets osteoclasts, not osteoblasts. In conjunction with standard diagnostic procedures, the more mechanistic data related to the adverse effects of ALN can also act as an applicable supplement for clinical judgment.

## Supplementary Material

Supplementary methods.

## Figures and Tables

**Table 1:** List of differentially expressed proteins (DEPs) with corresponding statistics.

Uniprot ID	Gene Symbol	Full Name	Log2 FC (ALN/ Ctrl)	Median P-Value	T Test P- Value	Adj. P
**Q86VN1**	VPS36	Vacuolar protein-sorting-associated protein 36	2.3697	0.0191	0.3002	0.0332
**Q9BXR6**	CFHR5	Complement factor H-related protein 5	1.5412	0.0306	0.0824	0.0106
**Q8N350**	CBARP	Voltage-dependent calcium channel beta subunit-associated regulatory protein	1.23	0.0776	0.0415	0.0129
**P48163**	ME1	NADP-dependent malic enzyme	1.2145	0.1359	0.0149	0.0103
**Q9ULH7**	MRTFB	Myocardin-related transcription factor B	1.1752	0.1301	0.0877	0.0397
**P14921**	ETS1	Protein C-ets-1	1.1566	0.1653	0.0497	0.0319
**Q9H0V9**	LMAN2L	VIP36-like protein	1.1446	0.013	0.1852	0.0136
**Q15427**	SF3B4	Splicing factor 3B subunit 4	1.0902	0.3234	0.0296	0.0487
**Q9H223**	EHD4	EH domain-containing protein 4	0.8995	0.113	0.1213	0.0463
**Q07021**	C1QBP	Complement component 1 Q subcomponent-binding protein, mitochondrial	0.8848	0.1071	0.1049	0.0388
**Q9UJW2**	TINAG	Tubulointerstitial nephritis antigen	0.8833	0.0976	0.1428	0.0474
**P56192**	MARS1	Methionine--tRNA ligase, cytoplasmic	0.8772	0.0039	0.1283	0.0037
**P08579**	SNRPB2	U2 small nuclear ribonucleoprotein B	0.8721	0.0322	0.2438	0.0361
**Q9H4B7**	TUBB1	Tubulin beta-1 chain	0.8644	0.0467	0.0944	0.0172
**Q9H2H8**	PPIL3	Peptidyl-prolyl cis-trans isomerase-like 3 (PPIase)	0.8257	0.1129	0.1055	0.0408
**P00439**	PAH	Phenylalanine-4-hydroxylase (PAH)	0.8024	0.0475	0.1114	0.0206
**Q5JTZ9**	AARS2	Alanine--tRNA ligase, mitochondrial	0.7071	0.0877	0.0325	0.0118
**Q9UKN8**	GTF3C4	General transcription factor 3C polypeptide 4	0.6861	0.0038	0.1761	0.0055
**Q92747**	ARPC1A	Actin-related protein 2/3 complex subunit 1A (SOP2-like protein)	0.6813	0.1877	0.0156	0.0158
**P99999**	CYCS	Cytochrome c	0.664	0.0456	0.1572	0.0283
**Q7Z2W4**	ZC3HAV1	Zinc finger CCCH-type antiviral protein 1	0.6468	0.1397	0.0465	0.0254
**Q9Y5M8**	SRPRB	Signal recognition particle receptor subunit beta	0.645	0.0983	0.1459	0.0486
**P62191**	PSMC1	26S proteasome regulatory subunit 4	0.6447	0.1717	0.0328	0.0243
**P43251**	BTD	Biotinidase (Biotinase)	0.6273	0.1495	0.0734	0.0392
**P08648**	ITGA5	Integrin alpha-5	0.6102	0.0891	0.1285	0.0398
**Q07955**	SRSF1	Serine/arginine-rich splicing factor 1	0.6084	0.0123	0.0315	0.0018
**P18754**	RCC1	Regulator of chromosome condensation	0.5984	0.1047	0.008	0.0048
**P62140**	PPP1CB	Serine/threonine-protein phosphatase PP1-beta catalytic subunit	-0.6001	0.1545	0.0111	0.0097
**Q9NX40**	OCIAD1	OCIA domain-containing protein 1	-0.601	0.0912	0.1301	0.041
**Q14244**	MAP7	Microtubule-associated protein 7	-0.6115	0.0198	0.1806	0.0178
**Q9Y572**	RIPK3	Receptor-interacting serine/threonine-protein kinase 3	-0.6157	0.0717	0.0554	0.0153
**Q06187**	BTK	Bruton tyrosine kinase	-0.6434	0.2767	0.0165	0.027
**Q8IW35**	CEP97	Centrosomal protein of 97 kDa	-0.6669	0.0989	0.1153	0.0394
**Q969G5**	CAVIN3	Caveolae-associated protein 3	-0.6891	0.0308	0.0337	0.0045
**O96033**	MOCS2	Molybdopterin synthase sulfur carrier subunit	-0.6914	0.0731	0.0987	0.0262
**P13798**	APEH	Acyl-peptide hydrolase	-0.6929	0.0508	0.239	0.0485
**Q9H3H3**	C11orf68	UPF0696 protein C11orf68	-0.7029	0.0819	0.1651	0.0471
**Q08431**	MFGE8	Milk fat globule-EGF factor 8	-0.7468	0.1464	0.0932	0.0466
**Q9NYJ8**	TAB2	TGF-beta-activated kinase 1	-0.7572	0.1619	0.0095	0.0092
**O95218**	ZRANB2	Zinc finger Ran-binding domain-containing protein 2	-0.8387	0.1273	0.054	0.0261
**Q93074**	MED12	Mediator of RNA polymerase II transcription subunit 12	-0.91	0.0239	0.2422	0.0291
**Q15047**	SETDB1	Histone-lysine N-methyltransferase SETDB1	-0.9105	0.2702	0.0382	0.0459
**P33241**	LSP1	Lymphocyte-specific protein 1	-0.9375	0.0021	0.242	0.0058
**Q9H3M7**	TXNIP	Thioredoxin-interacting protein	-0.9387	0.0348	0.086	0.0123
**P22307**	SCP2	Sterol carrier protein X	-1.0061	0.1877	0.0682	0.0465
**Q96A49**	SYAP1	Synapse-associated protein 1	-1.0203	0.0638	0.0634	0.0155
**Q5T1M5**	FKBP15	FK506-binding protein 15	-1.0953	0.0796	0.135	0.0379
**Q9NR77**	PXMP2	Peroxisomal membrane protein 2	-1.1041	0.397	0.0075	0.0284
**Q6P4R8**	NFRKB	Nuclear factor related to kappa-B-binding protein	-1.131	0.0431	0.1973	0.0347
**A6ND91**	ASPDH	Aspartate dehydrogenase domain-containing protein	-1.2357	0.0503	0.2253	0.0451
**Q9Y320**	TMX2	Thioredoxin-related transmembrane protein 2	-1.3223	0.1468	0.0342	0.0211
**Q9BRK0**	REEP2	Receptor expression-enhancing protein 2	-1.3431	0.0303	0.0738	0.0094
**Q9UHK6**	AMACR	Alpha-methylacyl-CoA racemase	-1.3986	0.0022	0.2013	0.0046
**P49407**	ARRB1	Beta-arrestin-1 (Arrestin beta-1)	-1.4064	0.1261	0.016	0.01
**Q92630**	DYRK2	Dual specificity tyrosine-phosphorylation-regulated kinase 2	-1.4443	0.0139	0.0418	0.0027
**Q9BRU9**	UTP23	rRNA-processing protein UTP23 homolog	-1.8162	0.0833	0.0247	0.009
**O14617**	AP3D1	AP-3 complex subunit delta-1	-1.9901	0.0471	0.0127	0.0029
**Q9C073**	FAM117A	Protein FAM117A (C/EBP-induced protein)	-3.5266	0.0051	0.308	0.015
