# Supplementary material for: Alendronate-induced Perturbation of the Bone Proteome and Microenvironmental Pathophysiology
Source: Int J Med Sci. 2021 Jul 23;18(14):3261–70. doi: 10.7150/ijms.61552 (PMC8364444; doi:10.7150/ijms.61552)
Supplement: Supplementary file 1 — Supplementary methods. [file ijmsv18p3261s1.pdf]

MATERIALS AND METHODS

Cell culture

Human osteoblast-like MG-63, SCC-9, SCC-15, and HUVEC cells were obtained from the American Type Culture Collection (ATCC) (Manassas, VA). The base medium for this cell line was ATCC-formulated Eagle's Minimum Essential Medium (EMEM) (Catalog No. 30-2003). To make the complete growth medium, the following components were added to the base medium: heat-inactivated fetal bovine serum (FBS) (Thermo Fisher Scientific, Waltham, MA) to a final concentration of 10%, and 1% penicillin and streptomycin (Thermo Fisher Scientific). HUVEC cells were cultured in Roswell Park Memorial Institute (RPMI)-1640 medium with 20% FBS and 60 µg/mL of endothelial cell growth supplement (BD Biosciences, San Jose, CA). The macrophage cell line, RAW 264.7, was purchased from Sigma Cells (St. Louis, MO, USA) and was cultured in Dulbecco's Modified Eagle Medium (DMEM) supplemented with 10% FBS, as instructed. All cells were kept in humidified incubators with 5% CO_2 at 37 °C. Culture media was changed after one day of subculturing and cells were passed again when there was 70%–80% confluence. All cells were regularly tested.

Antibodies and reagents

Antibodies against phospho-glycogen synthase kinase-3β (GSK-3β) (S9, inactive form), GSK3β, p-βcatenin (S45), β-catenin, p-EGFR (Y1068, active form), EGFR, and β-actin were obtained from Cell Signaling Technology. Antibodies against RIPK3, arrestin β1, and TGF-α were purchased from Novus Biologicals, LLC (Centennial, CO) and Abcam (Cambridge, UK). Horseradish peroxidase (HRP)-conjugated secondary antibodies were purchased from Cell Signaling Technologies. Reagents, including ALN, zoledronic acid (ZLN), clodronate (CLN), and bacterial lipopolysaccharide (LPS) (*Escherichia coli*, 0111: B4), were purchased from Sigma Chemical Corp. Mass spectrometry-grade reagents (column, buffer et al.) were purchased from Sigma. The Mouse Proteome Profiler Array was purchased from R&D Systems. The remaining chemical reagents used for biological, biochemical, and functional experiments were procured from Sigma Chemical Corp.

Quantitative proteomics

1) Sample preparation

For this study, we performed proteomics profiling as follows. Cell pellets were lysed in 8 M urea with 0.1% Rapigest in 50 mM Tris (pH 8.0) using sonication. The protein concentration was assessed via Pierce BCA Assay (Thermo Fisher Scientific, Waltham, MA, USA) and 50 µg of protein were aliquoted per sample for mass spectrometry (MS) analysis. Protein sulfydryl bonds were reduced with 10 mM ditriothreitrol (DTT) treatment for 30 mins at 37 ºC, and subsequently reduced cysteines were blocked with 100 mM iodoacetamide incubated for 30 mins at 25 ºC in the dark. A neutral pH between 7-8 was confirmed for each sample and trypsin was added at a ratio of 1:25 µg trypsin to total protein. Samples were digested overnight at 37 ºC and the resulting peptides were desalted on NEST C18 tips, according to the manufacturer's instructions. The peptides were then eluted using 2 x 100 µL of 50% acetonitrile / 0.1% formic acid. The resulting clean, eluted peptides were dried to completeness using a speed vacuum and stored at -80 ºC until MS acquisition.

Samples were resuspended in 0.1% formic acid with a 1:40 dilution of Biognosys iRT Reference Peptides (Biognosys, Schlieren Switzerland) at a concentration of 1 µg / ul. Next, 5 µL of peptide solution was injected into a 15 cm Phenomenex Omega Polar C18 3 um 100A 150 x 0.3 mm column and separated over 90 min with a gradient transitioning from 0-45% acetonitrile (buffer B) in 0.1% formic acid (buffer A) at a flow rate of 7 µl / min. Peptides were ionized by electrospray into a Thermo Fusion Lumos Mass Spectrometer operating in data independent acquisition mode.

The instrument cycled continuously between (a) an intact MS1 scan of all peptides between 400-1600 m/z in the orbitrap detector at a resolution of 120K with an accumulation time of 50 ms and target AGC of 400K, and (b) 40 subsequent MS2 scans systematically isolating all ions within 15 mz range intervals from 400-1000 m/z and analyzing high energy induced collision (CE 30%) induced fragments between 200-2000 m/z from each window in the orbitrap at 30K resolution, maximum injection time of 54 per scan and target AGC set to 500K. Total cycle time to progress through each MS1 and 40 MS2 scan series was 3 sec.

2) **Protein quantification and statistical analysis using mapDIA**
Data was analyzed based on the established workflows previously described(Parker et al. 2018; Parker et al. 2016). Briefly, peptides were identified using the openSWATH workflow(Rost et al. 2014), searched against the pan human library(Rosenberger et al. 2014) with decoy sequences appended for false discovery rate calculation using the pyprophet algorithm(Teleman et al. 2015). Peptides with no greater than 5% identified false discovery rate (FDR) across all samples were compiled into the final experimental results using the TRIC alignment algorithm(Rost et al. 2016). Following removal of non-proteotypic peptides (e.g., sequences matching more than one gene product from the Pan Human library), the final aligned results were analyzed using mapDIA software to select only high-quality performing fragments for quantification and to compile fragment level data into peptide and protein level abundance estimates (Teo et al. 2015). The mapDIA software was also used to perform pairwise comparisons between the ALN and control groups including adjustment for multiple testing effects to produce a comparison FDR, which filtered proteins with significant or non-significant differential abundance in response to ALN treatment. The MS proteomics data has been deposited to the PRIDE repository with the dataset identifier, PXD024585.

3) Identification of differentially expressed proteins (DEPs)
Proteins with more than 3 nonredundant peptides in each sample were selected. Further selection of proteins detected in at least 2 samples in the same group was done for statistical testing. Median difference test and Welch's t-test were performed separately, and the resulting two p-values were combined to compute adjusted p-values using Stouffer's method. The DEPs were identified based on an adjusted p-values<0.05 and absolute $\log_2$ fold-change (FC) ≧0.58.

4) Identification of known angiogenesis-associated genes
To identify genes associated with angiogenesis, functional enrichment analysis was performed using DAVID. Genes were surveyed against the following Gene Ontology Biological Processes (GOBP) terms: angiogenesis, positive regulation of angiogenesis, negative regulation of angiogenesis, and regulation of angiogenesis.

VEGF ELISA assay
To determine vascular endothelial growth factor (VEGF-A) levels of conditioned medium from MG-63 cells incubated with ALN, supernatants from cell culture were analyzed using the Human VEGF Quantikine ELISA Kit (R&D Systems, Minneapolis, Mass).

Cell proliferation and viability assays
MG-63 cells were grown onto 6-well plates at a density of 5 × 10^3 cells per mL for 24 h, and exposed to various concentrations of ALN to determine the optimum modeling concentration. In some experiments, 10 µM of ALN, ZLN, or CLN were used to treat MG-63, SCC-9, or SCC-15 cells. Cell numbers were evaluated after 6 hours of BP treatment. To determine the proliferation rate, the conditioned medium from MG-63 cells incubated with or without ALN-containing medium was used to culture HUVEC. Cell viability assays were performed using the 3-(4,5-dimethylthiazol-2-yl)-2,5-diphenyltetrazolium bromide (MTT) analysis, and the absorbance was

measured at a wavelength of 570 nm on a microplate reader, according to the manufacturer's instructions (Abcam, Cambridge, UK). For crystal violet staining, cells were stained with a 0.05% solution of crystal violet. After incubation at room temperature for 15 min, the wells were washed thoroughly with phosphate-buffered saline and fixed with 4% paraformaldehyde at room temperature for an additional 5 min. For quantitative analysis, a 10% acetic acid solution was used to dissolve the stained cells and absorbance at 570–590 nm was measured. All data are representative of 6 independent trials.

Western blotting

Cellular proteins from 10μM ALN-treated and control MG-63 cells were prepared using a 4% sodium dodecyl sulfate-containing lysis buffer. The protein concentration was measured using the Pierce 660nm Assay Kit (Thermo Fisher Scientific, Waltham, MA, USA). The cell lysates were mixed with a 4x protein sample loading buffer (10% SDS, 500mM DTT, 50% Glycerol, 500mM Tris-HCL and 0.05% bromophenol blue dye) (BioRad, Hercules, CA, USA) and heated for 10 min at 100 °C. After a brief centrifugation (13 000 × g) at 4 °C for 10 min, the protein concentration was quantified using the Bio-Rad Protein Assay (Bio-Rad, Hercules, CA, USA). Samples (50 μg) from all groups were subjected to a 12% sodium dodecyl sulfate PAGE and transferred onto nitrocellulose membranes (Bio-Rad, Hercules, CA, USA). These membranes were blocked with 5% skimmed milk at 37 °C for 60 min and then immersed with primary antibodies (1: 1000, Cell Siganling) at 4 °C overnight. After washing with 1x TBST (0.1% Tween-20, 10 mM Tris-base and 100 mM NaCl; pH 7) three times, the blots were incubated for 1 h with a horseradish peroxidase (HRP)-conjugated secondary antibody (1: 2000; Abcam). Following additional TBST washes, visualization of the protein bands was executed using an Enhanced Chemiluminescence Kit (ECL; Thermo Fisher Scientific, MA, USA) and Gel & Blot Imaging System and quantified using ImageJ.

Cytokine array

Cell lysates and conditioned media from RAW 264.7 macrophages were collected and analyzed using a cytokine array per standard provided protocols (R&D Systems, Minneapolis, MN, USA). Briefly, after mixing with a cocktail of biotinylated detection antibodies, the mixture was then incubated with the mouse cytokine array membrane. After washing several times to remove unbound materials on the membrane, streptavidin-HRP and chemiluminescent detection reagents were added. ImageJ was used to measure the intensities of dots.

Mineralization assay using Alizarin Red-S staining

The formation of calcium phosphate was quantified in MG-63 cells via Alizarin Red-S mineralization assay. After rinsing 3 times with PBS, cells were fixed in 4% (v/v) paraformaldehyde at room temperature for 20 min, and then stained with 2% Alizarin Red-S (pH 4.1–4.3) (Sigma-Aldrich, St. Louis, MO, US) for 15 min. Then, 1 mL of 100 mM cetylpyridinium chloride (Sigma-Aldrich, St. Louis, MO, US) was added to dissolve the dried Alizarin Red-S staining. Optical density was detected at an absorbance of 562 nm.

Statistical analysis

All experiments were repeated at least three times, most of cases 6 times, with independent treatments. Each of the experiments did not show significantly different results across replications. Statistical analyses were conducted using GraphPad Prism, version 7.03 (GraphPad Software Inc., La Jolla, CA). Mean values from technical replicates were used for statistical analyses, and all data were presented as the mean ± standard deviation (SD). A one-way analysis of variance (ANOVA) or Student's t-test was conducted to compare the groups of data. Differences were considered statistically significant when $P < 0.05$.
